# Trichloracetic Acid as a Solvent of Necrosis

**Published:** 1897-09

**Authors:** H. W. Allwine

**Affiliations:** Omaha, Neb.


ARTICLE IV.

### TRICHLORACETIC ACID AS A SOLVENT OF NECROSIS.

BY H. W. ALLWINE, D. D. S., OMAHA, NEB.

Much has b[illegible]n and is written on the treatment of pyorrhea alveolar[illegible] but it is not my intention to discuss the disease in this [illegible]nnection; but on account of the uniformly satisfactory [illegible]sults reached by using trichloracetic acid in the treatm[illegible] of pyorrhea, to earnestly advocate it. By a properly co[illegible]ued use of this acid at regular periods, and an antiseptic [illegible]uthwash at all times, I have obtained better results tha[illegible]y any other treatment.

Knowing that the treatment of pyorrhea alveolaris requires medicaments which dissolve calcareous matter as well as break down diseased soft tissue and stimulate new granulations, I determined to give it a thorough test in treating a case of necrosis that was sent to me, which I now describe. There is no reason, in my mind, why treatment of the two diseases should not be very similar.

Last June Mrs. B. consulted a physician concerning a trouble apparently over, or at the symphysis of the inferior maxillary, and the case was placed in my hands by him. At that time the muscles about the chin were very tender and painful to the least motion, swollen, and of a dark blue color on the outside, as well as about the roots of the teeth. A fistula had formed over the root of the left lateral, from which pus exuded. This tooth was also quite sore to the touch.

I believed the pulp of this tooth was dead, and proceeded to drill into the lingual surface of the tooth. Before proceeding far I discovered my error. I forced mild antiseptics into the fistula, applied a counter-irritant, and dismissed the patient, that I might give the case careful study, In a few days the lady returned. It being a clear day, I noticed a very slight discoloration of the two centrals. These teeth were not in the least sore, but on drilling into them I found the pulps dead. The probe passed through the fistula indicated the unmistakable roughness of necrosis to the touch of the physician and surgeon, and to my own.

The quickest way out of the difficulty, I thought, was to make an incision and remove the cause. I also suggested to the patient the possibility of dissolving the dead structure with acid, though it might require a long time. She much preferred a fair trial of the latter method, so about once in two weeks I injected a 10 per cent. solution of trichloracetic acid. Twice a week a mild antiseptic was passed into the left central, through the fistula. The apex of the right central was open nicely, but I could force nothing through the fistula from this tooth. At each visit I packed the canals with cotton saturated with mild antiseptics, and carefully sealed the cavities. I thus treated the case nearly three months. Indications being very favorable, I packed the canals, sealed the cavities, and told the patient to return in two weeks. At this time a very little healthy serum was found in the left central. I treated the case as before, and told the patient to return two weeks later. Then the conditions were such as I wished to have, and I proceeded to put in the fillings. This was done the latter part of September. December 11 I saw the case. The condition was entirely satisfactory, and I believe a permanent cure was effected.

I will state briefly the history of the case. In February, 1895, Mrs. B. noticed a slight neuralgic pain in the chin, the muscles inclining to be a little stiff. This continued to grow worse, and the latter part of December, 1895, a fistula formed, as above described, and pus continued to discharge. She herself made various local applications, but the condition continuing to grow worse, she became anxious and sought advice, as previously stated.

It is very desirable that causes should be ascertained. In this case I do not find one that to me is satisfactory. Mrs. B. is 32 years of age, of mixed temperament, the bilious predominating, and of very regular habits. She is a regular, systematic pedestrian. Her health is and has been good. No calcareous nodules or deposits were found in the root canals. No injury of any kind had been received in the vicinity of the trouble. Prior to coming here from New York State her energies were taxed somewhat, on account of sickness in the family. I should not look to the climatic change as the cause. November, 1894, shortly before the appearance of disturbance, there was suppressed menstruation, which she thought at the time might be impregnation, but it was not. Was the cause a combination of the last three conditions and changes?—*Atlanta Dental Journal.*

